# Supplementary material for: Meta-Analysis and Gene Set Enrichment Relative to ER Status Reveal Elevated Activity of MYC and E2F in the “Basal” Breast Cancer Subgroup
Source: PLoS One. 2009 Mar 9;4(3):e4710. doi: 10.1371/journal.pone.0004710 (PMC2650420; doi:10.1371/journal.pone.0004710)
Supplement: Dataset S1 — GSEA results from first screen. The file contains a zipped archive of the HTML results of the second GSEA screen. Reports for enriched or depleted datasets may be accessed from the “index.html” link within the my_analysis.GseaPreranked.1219975950625.rpt.zip ZIP archive. Enrichment plots are available for each of the 50 most enriched and depleted gene sets. (13.32 MB ZIP) [file pone.0004710.s001.zip › my_analysis.GseaPreranked.1219975950625.rpt.zip!//CHR14Q23.html]

Details for gene set CHR14Q23[GSEA]

|  || Dataset | zSco.ERneg.to.ERpos\_collapsed |
| Phenotype | NoPhenotypeAvailable |
| Upregulated in class | na\_neg |
| GeneSet | CHR14Q23 |
| Enrichment Score (ES) | -0.530385 |
| Normalized Enrichment Score (NES) | -1.8518518 |
| Nominal p-value | 0.0 |
| FDR q-value | 0.026590241 |
| FWER p-Value | 0.602 |
Table: GSEA Results Summary

  

Fig 1: Enrichment plot: CHR14Q23      
 Profile of the Running ES Score & Positions of GeneSet Members on the Rank Ordered List

  

| PROBE | GENE SYMBOL | GENE\_TITLE | RANK IN GENE LIST | RANK METRIC SCORE | RUNNING ES | CORE ENRICHMENT || 1 | PLEK2 |  |  | 3721 | 1.614 | -0.2347 | No |
| 2 | GPR135 |  |  | 5102 | 1.115 | -0.3074 | No |
| 3 | ATP6V1D |  |  | 5604 | 0.943 | -0.3187 | No |
| 4 | PLEKHG3 |  |  | 6277 | 0.723 | -0.3491 | No |
| 5 | ZBTB1 |  |  | 7094 | 0.452 | -0.3978 | No |
| 6 | RAD51L1 |  |  | 7218 | 0.412 | -0.3955 | No |
| 7 | DHRS7 |  |  | 7249 | 0.401 | -0.3866 | No |
| 8 | SGPP1 |  |  | 7265 | 0.398 | -0.3766 | No |
| 9 | C14ORF135 |  |  | 7370 | 0.359 | -0.3744 | No |
| 10 | PSMA3 |  |  | 7737 | 0.218 | -0.3958 | No |
| 11 | RDH11 |  |  | 8046 | 0.088 | -0.4165 | No |
| 12 | KIAA0586 |  |  | 8731 | -0.183 | -0.4629 | No |
| 13 | ZBTB25 |  |  | 9446 | -0.537 | -0.5016 | Yes |
| 14 | MAX |  |  | 9664 | -0.653 | -0.4996 | Yes |
| 15 | PRKCH |  |  | 9700 | -0.668 | -0.4836 | Yes |
| 16 | MPP5 |  |  | 10323 | -1.042 | -0.5013 | Yes |
| 17 | RAB15 |  |  | 10454 | -1.126 | -0.4796 | Yes |
| 18 | FNTB |  |  | 10625 | -1.265 | -0.4570 | Yes |
| 19 | SIX1 |  |  | 11441 | -1.979 | -0.4630 | Yes |
| 20 | GPHN |  |  | 11980 | -2.540 | -0.4325 | Yes |
| 21 | VTI1B |  |  | 12342 | -3.009 | -0.3756 | Yes |
| 22 | ZFYVE26 |  |  | 12515 | -3.287 | -0.2967 | Yes |
| 23 | SLC38A6 |  |  | 12847 | -3.994 | -0.2100 | Yes |
| 24 | MNAT1 |  |  | 12891 | -4.114 | -0.0983 | Yes |
| 25 | TMEM30B |  |  | 13057 | -4.670 | 0.0198 | Yes |
Table: GSEA details [plain text format]

  

Fig 2: CHR14Q23: Random ES distribution      
 Gene set null distribution of ES for **CHR14Q23**
